# The effectiveness of COVID-19 vaccine in the prevention of post-COVID conditions: a systematic literature review and meta-analysis of the latest research

**DOI:** 10.1017/ash.2023.447

**Published:** 2023-10-13

**Authors:** Alexandre R. Marra, Takaaki Kobayashi, Gustavo Yano Callado, Isabele Pardo, Maria Celidonio Gutfreund, Mariana Kim Hsieh, Vivian Lin, Mohammed Alsuhaibani, Shinya Hasegawa, Joseph Tholany, Eli N. Perencevich, Jorge L. Salinas, Michael B. Edmond, Luiz Vicente Rizzo

**Affiliations:** 1 Department of Internal Medicine, University of Iowa Carver College of Medicine, Iowa City, IA, USA; 2 Faculdade Israelita de Ciências da Saúde Albert Einstein, Hospital Israelita Albert Einstein, São Paulo, SP, Brazil; 3 Center for Access & Delivery Research & Evaluation (CADRE), Iowa City Veterans Affairs Health Care System, Iowa City, IA, USA; 4 Department of Pediatrics, King Faisal Specialist Hospital & Research Centre, Riyadh, Saudi Arabia; 5 Stanford University, Stanford, CA, USA; 6 West Virginia University School of Medicine, Morgantown, WV, USA

## Abstract

**Objective:**

We performed a systematic literature review and meta-analysis on the effectiveness of coronavirus disease 2019 (COVID-19) vaccination against post-COVID conditions (long COVID) among fully vaccinated individuals.

**Design:**

Systematic literature review/meta-analysis.

**Methods:**

We searched PubMed, Cumulative Index to Nursing and Allied Health, EMBASE, Cochrane Central Register of Controlled Trials, Scopus, and Web of Science from December 1, 2019, to June 2, 2023, for studies evaluating the COVID-19 vaccine effectiveness (VE) against post-COVID conditions among fully vaccinated individuals who received two doses of COVID-19 vaccine. A post-COVID condition was defined as any symptom that was present four or more weeks after COVID-19 infection. We calculated the pooled diagnostic odds ratio (DOR) (95% confidence interval) for post-COVID conditions between fully vaccinated and unvaccinated individuals. Vaccine effectiveness was estimated as 100% x (1-DOR).

**Results:**

Thirty-two studies with 775,931 individuals evaluated the effect of vaccination on post-COVID conditions, of which, twenty-four studies were included in the meta-analysis. The pooled DOR for post-COVID conditions among fully vaccinated individuals was 0.680 (95% CI: 0.523–0.885) with an estimated VE of 32.0% (11.5%–47.7%). Vaccine effectiveness was 36.9% (23.1%–48.2%) among those who received two doses of COVID-19 vaccine before COVID-19 infection and 68.7% (64.7%–72.2%) among those who received three doses before COVID-19 infection. The stratified analysis demonstrated no protection against post-COVID conditions among those who received COVID-19 vaccination after COVID-19 infection.

**Conclusions:**

Receiving a complete COVID-19 vaccination prior to contracting the virus resulted in a significant reduction in post-COVID conditions throughout the study period, including during the Omicron era. Vaccine effectiveness demonstrated an increase when supplementary doses were administered.

## Background

In the last three years, extensive research has demonstrated the safety and efficacy of COVID-19 (Coronavirus Disease 2019) vaccines.^1,2^ These vaccines have played a critical role in reducing mortality and hospitalization rates.^3,4^ Furthermore, studies have confirmed the value of additional COVID-19 vaccine doses in sustaining immunization effectiveness and guarding against emerging variants.^5,6^ However, post-COVID conditions, commonly known as long COVID, have become a significant concern as growing evidence suggests that a substantial number of individuals continue to experience persistent symptoms and complications long after the acute phase of the illness.

The Centers for Disease Control and Prevention (CDC) defines post-COVID conditions as a vast range of ongoing health problems (e.g., cardiovascular, respiratory, and neuropsychiatric symptoms) that can last for more than 4 weeks after an individual has been infected by severe acute respiratory coronavirus virus 2 (SARS-CoV-2) virus.^7^ These conditions can significantly impact individuals’ quality of life, and daily functioning, and pose a considerable burden on the healthcare system. As of January 2023, 28% of individuals who had a previous COVID-19 infection experienced post-COVID conditions.^8^ A systematic review published previously demonstrated that receiving at least one dose of Pfizer/BioNTech, Moderna, AstraZeneca, or Janssen vaccines could prevent the occurrence of long COVID symptoms.^9^

As vaccination campaigns have progressed, the majority of people have received more than one dose of COVID-19 vaccines. However, their effectiveness in preventing post-COVID conditions among fully vaccinated individuals remains an unresolved question.^10,11^ The vaccine effectiveness (VE) against post-COVID symptoms might vary depending on the number of vaccine doses people have received. Hence, our objective was to conduct a literature review on the effectiveness of COVID-19 vaccines, specifically examining the impact of receiving two or more doses of these vaccines in preventing post-COVID conditions. By pooling the findings of published studies, we aimed to provide more accurate estimates of vaccine effectiveness.

## Methods

### Systematic literature review and inclusion and exclusion criteria

This review was conducted according to the Preferred Reporting Items for Systematic Reviews and Meta-Analysis statement^12^ and the Meta-analysis of Observational Studies in Epidemiology guidelines^13^ and was registered on Prospero (https://www.crd.york.ac.uk/PROSPERO/) on 5/24/2023 (registration number CRD42023429149). Institutional Review Board approval was not required. Inclusion criteria for studies in this systematic review were as follows: original research manuscripts; published in peer-reviewed, scientific journals; involved fully vaccinated (at least two doses of COVID-19 vaccines [mRNA, or vectorial or inactivated viral vaccine], with exception of one dose for Janssen [Ad26.COV2.S] vaccine), and unvaccinated individuals; evaluated the long-term effectiveness of the COVID-19 vaccine; and observational study design. Post-COVID condition (also known as long COVID) was defined as a wide range of health symptoms that are present four or more weeks after COVID-19 infection.^7^ The literature search included studies from December 1, 2019 to June 2, 2023. Editorials, commentaries, reviews, study protocols, studies analyzing one dose of the COVID-19 vaccine, and studies in the pediatric population were excluded. Studies comparing partially vaccinated (one dose of COVID-19 vaccine) with unvaccinated individuals were excluded. Studies without a comparison between vaccinated and unvaccinated individuals (or other vaccinated control groups) were also excluded.

### Search strategy

**Figure 1.**
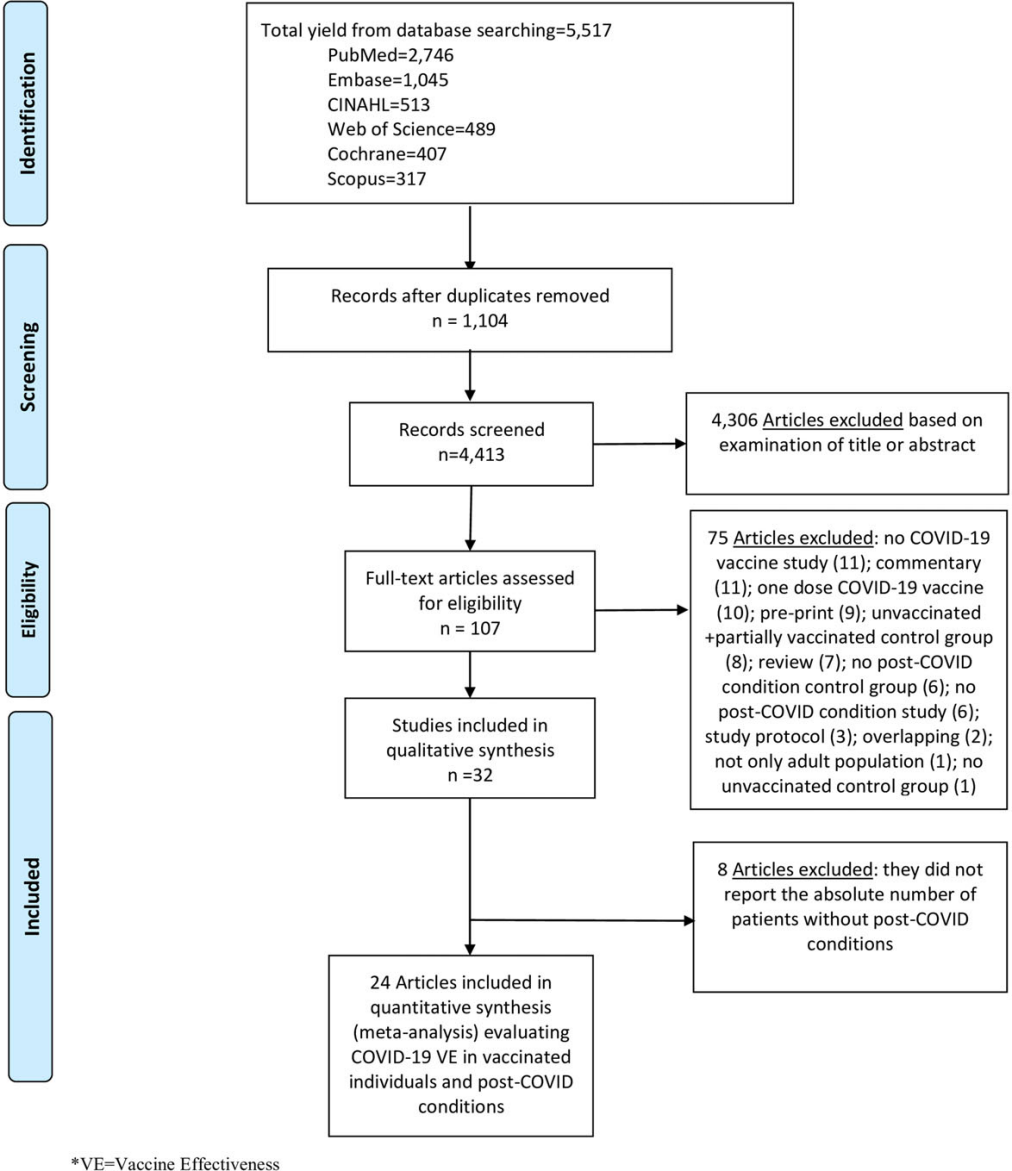
Literature search for articles on the COVID-19 vaccine effectiveness in post-COVID conditions.

We performed literature searches in PubMed, Cumulative Index to Nursing and Allied Health, Embase (Elsevier Platform), Cochrane Central Register of Controlled Trials, Scopus (which includes EMBASE abstracts), and Web of Science. The entire search strategy is described in Supplementary Appendix 1. After applying the exclusion criteria, we reviewed 107 papers, out of which 32 met the inclusion criteria and were included in the systematic literature review (Figure 1).

### Data abstraction and quality assessment

Titles and abstracts of all articles were screened to assess whether they met the inclusion criteria. Abstract screening was performed by one reviewer (ARM). Of ten independent reviewers (ARM, GYC, IP, JT, MA, MCG, MH, SH, TK, and VL), two independently abstracted data for each article using a standardized abstraction form. Reviewers resolved disagreements by consensus.

The reviewers abstracted data on study design, population and location, study period (months) and calendar time, demographic and characteristics of participants, and the types of COVID-19 vaccine if available. Post-COVID conditions were considered the primary outcome to calculate VE after at least two doses of a COVID-19 vaccine. Eleven corresponding authors were contacted for additional information, and four were able to provide additional information regarding the number of individuals with and without post-COVID conditions in both fully vaccinated and unvaccinated groups.^4–17^ Risk of bias was assessed using the Downs and Black scale.^18^ Reviewers answered all original questions from this scale except for question #27 (a single item on the Power subscale scored 0–5), which was changed to a yes or no. Two authors performed component quality analysis independently, reviewed all inconsistent assessments, and resolved disagreements by consensus.^19^

### Statistical analysis

To perform a meta-analysis of the extracted data, we calculated the pooled diagnostic odds ratio (DOR) for post-COVID conditions between fully vaccinated and unvaccinated individuals. Vaccine effectiveness was estimated as 100% x (1-DOR). We performed stratified analyses by the timing of the COVID-19 vaccine (i.e., those with COVID-19 vaccines before or after COVID-19 diagnosis, those with COVID-19 vaccines before COVID-19 diagnosis, those with COVID-19 vaccines after COVID-19 diagnosis, and those with COVID-19 vaccines before COVID-19 diagnosis during the Omicron variant era), and between those vaccinated with a first booster dose and unvaccinated individuals. We performed statistical analysis using R version 4.1.0 with mada package version 0.5.8.^20^ Analogous to the meta-analysis of the odds ratio methods for the DOR, an estimator of random-effects model following the approach of DerSimonian and Laird is provided by the mada package.^20^ For our meta-analysis of VE estimates against post-COVID conditions, we used a bivariate random-effects model, adopting a similar concept of performing the diagnostic accuracy. This enabled simultaneous pooling of sensitivity and specificity with mixed-effect linear modeling while allowing for the trade-off between them.^21,22^ Heterogeneity between studies was evaluated with I^2^ estimation and the Cochran Q statistic test. Publication bias was assessed using the Egger test with R version 4.1.0 with metafor package^23^.

## Results

### Characteristics of included studies

Thirty-two studies met the inclusion criteria^14–17,24–51^ and were included in the final review (Table 1). All studies were non-randomized^14–17,24–51^; of these, fourteen were retrospective cohort studies,^15,25,29–31,35,36,40,44–46,48,49,51^ nine were prospective cohort studies^14,16,28,32,34,37,38,41,50^, five were cross-sectional studies,^17,24,42,43,47^ and four were case-control studies.^26,27,33,39^ One of the cross-sectional studies was also within a prospective cohort study.^17^ More than half of these studies (22 out of 32) evaluated the Pfizer/BioNTech vaccine.^14–17,24–26,29–31,36–39,41–45,48–50^ Sixteen analyzed the Moderna vaccine,^15–17,25,26,29,31,36,41–45,48–50^ twelve analyzed the Janssen vaccine,^15,16,25,31,37,39,43–45,48–50^ ten analyzed the AstraZeneca vaccine,^15,16,24,26,28,29,33,39,44,48^ two the CoronaVac vaccine,^14,39^ one the Covaxin vaccine,^28^ one the Sinopharm vaccine,^33^ and one analyzed the Gamaleya vaccine.^15^ There were no published studies that evaluated post-COVID conditions as an outcome of bivalent COVID-19 vaccines.

**Table 1.** Summary of characteristics of studies included in the systematic literature review

First author, year, location, Study design, Study period in # of months and [dates]	COVID-19 vaccine, COVID-19 vaccine before COVID-19 infection	Participants (n) and characteristics	Post-COVID condition		Post-COVID condition		Post-COVID condition definition	Symptoms included in post-COVID condition studies	Benefit of COVID-19 vaccines to decrease post-COVID condition symptoms	D&B score (max= 28)
			Fully vaccinated	Control group [unvaccinated]	Vaccinated 1^st^ booster dose	Vaccinated 2^nd^ booster dose				
Al-Aly, 2022, USA Retrospective cohort study 10 [Jan 2021–Oct 2021]	Pfizer/BioNTech, Moderna, and Janssen Yes	33,940 fully vaccinated participants [U.S. Department of Veterans Affairs database] compared to several controls, including 113,474 unvaccinated participants with previous COVID-19 infection	33,940 [NR absolute numbers of long COVID in vaccinated group]	113,474 [NR absolute numbers of long COVID in unvaccinated group]	–	–	Long duration of COVID-19 symptoms ≥4 weeks	General: fatigue, post-exertional malaise; -Respiratory and heart: shortness of breath, chest pain, and fast beating or pounding heart; -Neurological: difficult thinking or concentrating (“brain fog”), headache, change in smell or taste, dizziness or lightheadedness, and pins-and-needles feelings; -Digestive: abdominal pain, diarrhea; -Other: joint or muscle pain	Yes. Compared to people with COVID-19 infection who were unvaccinated (n= 113,474), people with fully vaccination exhibited lower risks of post-COVID condition (HR = 0.85, 95% CI: 0.82, 0.89)	21
Alghamdi, 2022, Saudi Arabia Cross-sectional study 3 [Apr 2021–Jul 2021]	Pfizer/BioNTech, and AstraZeneca NR	2,218 individuals were recruited using social media	1,237 [NR absolute numbers of long COVID in vaccinated group]	132 [NR absolute numbers of long COVID in unvaccinated group] ]	–	–	Persistent neuropsychiatric disorders and conditions affecting the peripheral nerves from 6 months after COVID-19 infection	-Neurological: difficulty thinking or concentrating (“brain fog”), change in smell or taste, depression, and tinnitus sleeping disorders;	No. Post-COVID neuropsychiatric symptoms were present in considerable percentages of the study participants with SARS-CoV-2 infection, persisting for >6 months in up to 7.6% of the participants.	15
Antonelli, 2022 (1), UK Case-control study 7 [Dec 2020–Jul 2021]	Pfizer/BioNTech, Moderna, and AstraZeneca Yes	1,240,009 participants [COVID Symptoms Study app users] reported 1^st^ vaccine dose (6,030 [0.5% tested positive for SARS-CoV-2], and 971,504 reported 2^nd^ dose (2,370 [0.2% tested positive for SARS-CoV-2]	2,370 [NR absolute numbers of long COVID in vaccinated group]	2,370 [NR absolute numbers of long COVID in unvaccinated group]	–	–	Long duration of COVID-19 symptoms ≥4 weeks	-General: fatigue, post-exertional malaise, fever; -Respiratory and heart: shortness of breath, cough, chest pain, and fast beating or pounding heart; -Neurological: difficult thinking or concentrating (“brain fog”), headache, mood changes, change in smell or taste, dizziness or lightheadedness, and pins-and-needles feelings; -Digestive: abdominal pain, diarrhea; -Other: joint or muscle pain	Yes. Compared with unvaccinated individuals, after their second dose of COVID-19 vaccine, individuals were less likely to have prolonged illness (symptoms ≥28 d).	21
Antonelli, 2022, (2), UK Case-control study 7.5 [Jun 2021–Mar 2022]	NR Yes	97,364 participants [COVID Symptoms Study app users]. Among Omicron cases, 2501 (4·5%) of 56,003 people experienced post-COVID condition and, among Delta cases, 4,469 (10·8%) of 41,361 people experienced post-COVID condition	NR	NR	–	–	Long duration of COVID-19 symptoms ≥4 weeks	NR	Yes. There was a reduction in odds of post-COVID conditions with the Omicron variant versus the Delta variant of 0·24–0·50 depending on age and time since vaccination.	20
Arjun, 2022, India Prospective cohort study 6 [Apr 2021–Sep 2021]	AstraZeneca, and Covaxin NR	487 and 371 participants at 4 weeks and 6 months of follow-up, respectively. The incidence of post-COVID was 29.2%, and 9.4% in 4 weeks and 6 months of follow-up, respectively.	287 [98 with long COVID]	119 [24 with long COVID]	–	–	Long duration of COVID-19 symptoms ≥4 weeks	-General: fatigue; -Respiratory and heart: shortness of breath, cough, and fast beating or pounding heart; -Neurological: difficult thinking or concentrating (“brain fog”), anxiety, depression, and change in smell or taste;	No. Two doses of COVID-19 vaccination had higher odds of developing post-COVID condition.	23
Ayobkhani, 2022, UK Retrospective cohort study 20 [Apr 2020–Nov 2021]	Pfizer/BioNTech, Moderna, and AstraZeneca Yes	Of 3,333 eligible participants [UK COVID-19 Infection Survey] who were fully vaccinated before their first COVID-19 infection, 3,090 (92.7%) were 1:1 matched to participants who were unvaccinated when infected (from a pool of 9,854 potential control participants). Post-COVID condition symptoms was reported by 9.5% and 14.6% of fully vaccinated and unvaccinated individuals, respectively.	3,090 [294 with long COVID]	3,090 [452 with long COVID]	–	–	Long duration of COVID-19 symptoms ≥12 weeks	NR	Yes. COVID-19 vaccination at least 2 weeks before COVID-19 infection was associated with a 41% decrease in the odds of developing post-COVID condition symptoms at least 12 wk later, relative to not being vaccinated when infected.	20
Azzolini, 2022 Italy Retrospective cohort study 26 [Mar 2020–Apr 2022]	Pfizer/BioNTech Yes	2,560 participants [739 individuals (29%) had COVID-19 infection (89 asymptomatic) of whom 229 (31%) had post-COVID condition	46 [8 with long COVID]	421 [176 with Long COVID]	262 [42 with Long COVID]	–	Long duration of COVID-19 symptoms ≥4 weeks	General: fatigue, post-exertional malaise; -Respiratory and heart: shortness of breath, chest pain, and fast beating or pounding heart; -Neurological: difficult thinking or concentrating (“brain fog”), headache, change in smell or taste, dizziness or lightheadedness, and pins-and-needles feelings; -Digestive: abdominal pain, diarrhea; -Other: joint or muscle pain	Yes. Two or three doses of COVID-19 vaccine compared with unvaccinated individuals were associated with lower post-COVID condition prevalence.	21
Ballouz, 2023, Switzerland Retrospective cohort study 19 [Aug 2020–Feb 2022]	Pfizer/BioNTech, Moderna, and Janssen Yes	1,350 participants from two different cohorts [1,045 Zurich and 305 Corona Immunitas with 6 mo of follow-up]. Overall, 25.3% (*n* = 264) of individuals infected with Wildtype SARS-CoV-2, 17.2% (*n* = 17) of Delta-infected, and 13.1% (*n* = 27) of Omicron-infected individuals had post-COVID condition 6 months after COVID-19 infection.	232 [28 with long COVID]	1,114 [279 with long COVID]	171 [19 with long COVID]	–	The presence of symptoms within 6 months after COVID-19 infection	General: tiredness/fatigue; Respiratory: dyspnea, cough, chest pain, palpitations; Neurological: attention disorders, memory impairment (“brain fog”), headache; anosmia/hyposmia, dysgeusia, anxiety, dizziness, sensitive disorders, and sleep problems; Digestive: abdominal pain, nausea, and diarrhea; -Other: joint or muscle pain	Yes. The presence of either Omicron variant infection or prior vaccination appears to decrease the risk of post-COVID condition (OR, 0.42; 95% CI, 0.24-0.68).	20
Brunvoll, 2023, Norway Prospective cohort study 18 [Nov 2020–Jan 2021 (3 mo) up to 15 months after the COVID-19 test]	NR No	1,420 participants had a positive COVID-19 test between the third and fourth follow-up questionnaires, of which 1,060 were unvaccinated, and 360 were vaccinated with a breakthrough infection.	360 [NR absolute numbers of long COVID in vaccinated group]	1,060 [NR absolute numbers of long COVID in unvaccinated group]	–	–	Long duration of COVID-19 symptoms ≥12 weeks	General: tiredness/fatigue; Respiratory: dyspnea; Neurological: attention disorders, memory impairment (“brain fog”), anosmia/hyposmia, and dysgeusia;	Yes. COVID-19 vaccines offered minor protection against post-COVID conditions, although fewer memory problems were reported among the vaccinated than the unvaccinated participants.	20
El Otmani 2022, Morocco Case-control study 2 [Feb 2021–April 2021]	AstraZeneca and Sinopharm No	236 participants (118 COVID-19 infection and 118 matched controls) in an online survey. Post-COVID condition prevalence was 47.7%	63 [31 with long COVID]	55 [25 with long COVID]	–	–	Long duration of COVID-19 symptoms ≥12 weeks	General: fever, chills, and asthenia; Respiratory: dyspnea, cough, chest pain, and palpitations; Neurological: attention disorders, memory impairment (“brain fog”), headache, anosmia/hyposmia, tinnitus, and dysgeusia, anxiety, dizziness, and sensitive disorders; Digestive: abdominal pain, nausea, and diarrhea; Other: itching, anorexia, arthralgia, and myalgia	No. COVID-19 vaccination was not associated with the risk of post-COVID condition.	18
Emecen, 2023, Turkey Prospective cohort study 6 [Nov 2020–May 2021]	Pfizer/BioNTech, and Coronavac Yes	5,610 participants [telephone interview based COVID-19 follow-up] reported post-COVID condition, 37%, 21.8%, and 18.2% for the first, third, and sixth months.	120 [4 with long COVID]	4,041 [790 with long COVID]	–	–	The presence of symptoms within 6 months after COVID-19 infection	General: tiredness/fatigue; Respiratory: dyspnea, cough, chest pain, palpitations; Neurological: attention disorders, memory impairment (“brain fog”), headache; anosmia/hyposmia, dysgeusia, anxiety, dizziness, sensitive disorders, and sleep problems; Digestive: abdominal pain, nausea, and diarrhea; -Other: joint or muscle pain	Yes. Completion of the primary vaccine series prior to COVID-19 infection was associated with diminished risk for post-COVID condition (OR, 0.53; 95% CI, 0.40-0.72).	20
Hajjaji, 2022, France Prospective cohort study 16 [Apr 2020–Aug 2021]	NR NR	2,116 participants with cancer in a 2nd cohort study, being 168 individuals with COVID-19 infection [37 with long COVID and 131 with no post-COVID condition].	44 [7 with long COVID]	46 [11 with long COVID]	63 [NR absolute numbers of long COVID in booster dose group].	–	The presence of symptoms within 6 months after COVID-19 infection.	-General: fatigue, fever; -Respiratory and heart: shortness of breath, cough, chest pain, and fast beating; -Neurological: headache, mood changes, change in smell or taste, difficulty in concentrating, dizziness on standing, and asleep problems -Digestive: abdominal pain, and diarrhea; -Other: joint or muscle pain, rash	No. Vaccination was not associated with less post-COVID condition symptoms, suggesting that vaccines may not confer protection against COVID-19’s longer-term burden in cancer patients.	19
Hastie, 2022, Scotland Retrospective cohort study 21 [Mar 2020–Nov 2021]	NR NR	62,957 participants [33,281 (52.9%) infected and 29,676 (47.1%) never infected; of the 31,486 symptomatic COVID-19 infection, 1,856 (6%) had not recovered and 13,350 (42%) only partially.	NR	NR	–	–	Long duration of COVID-19 symptoms ≥12 weeks	-General: fatigue, fever; -Respiratory and heart: shortness of breath, cough, chest pain, and fast beating; -Neurological: headache, mood changes, change in smell or taste, difficulty in concentrating, dizziness on standing, and sleep problems -Digestive: abdominal pain, diarrhea; -Other: joint or muscle pain, rash	Yes. Compared to unvaccinated people, patients vaccinated prior to symptomatic infection were less likely to report persistent symptoms.	21
Hernandez-Aceituno 2023, Spain Retrospective cohort study 18 [Jan 2021–Jun 2022]	Pfizer/BioNTech, Moderna Janssen, AstraZeneca, Gamaleya Yes	296 adult patients hospitalized for COVID-19 with genomic sequencing information	219 [94 with long COVID]	77 [25 with long COVID]	NR	–	Long duration of COVID-19 symptoms ≥4 weeks	-General: fatigue, fever; -Respiratory and heart: shortness of breath, cough, chest pain, and fast beating; -Neurological: headache, mood changes, change in smell or taste, sleep problems, difficulty in concentrating, and dizziness; -Digestive: abdominal pain, diarrhea; nausea/anorexia; -Other: joint or muscle pain	No. Vaccination was not associated with less post-COVID condition symptoms. The Omicron variant was associated with significantly lower odds of developing post-COVID-conditions.	22
Ioannou, 2022, USA Retrospective cohort study 23 [Feb 2020–Dec 2021]	Pfizer/BioNTech, and Moderna Yes	198,601 participants [171 medical centers throughout the country; with 13.5% reported post-COVID condition]	2,447 [263 with long COVID]	58,693 [6,811 with long COVID]	–	–	Long duration of COVID-19 symptoms ≥12 weeks	NR	Yes. There is a protective effect of COVID-19 vaccination against developing post-COVID condition symptoms (adjusted OR, 0.78; 95% CI, 0.68-0.90).	21
Jassat, 2023, South Africa Prospective cohort study NR	Pfizer/BioNTech and Janssen Yes [and after COVID-19 infection too]	3,700 participants [identified through the national case list, and through Daily Hospital Surveillance (DATCOV); 46.7% of hospitalized and 18.5% of nonhospitalized participants with >= 1 persistent symptoms at 6 mo]	Any time: 2,535 [1,021 with Long COVID]; Before COVID-19 infection:731 [175 with long COVID]; After COVID-19 infection:1,804 [846 with Long COVID	Any time: 1,146 [398 with long COVID]; Before COVID-19 infection:1,146 [398 with long COVID]; After COVID-19 infection:1,146 [398 with long COVID	–	–	Long duration of COVID-19 symptoms ≥12 weeks	-General: fatigue; -Respiratory: shortness of breath, cough, and nasal congestion; -Neurological: headache, lack of concentration, and loss of smell; -Other: muscle pain	No. There was no difference in persistent symptoms in vaccinated and unvaccinated participants.	22
Kahlert, 2023, Switzerland Cross-sectional study [within prospective cohort] 2 [May 2022–Jun 2022]	Pfizer/BioNTech and Moderna Yes [and after COVID-19 infection too]	2,912 participant (post- COVID condition were more frequent after wild-type infection, after Alpha/Delta infection, and after Omicron BA.1 infections versus uninfected controls	Any time: 1,352 [373 with long COVID]; Before COVID-19 infection:1,088 [274 with long COVID]; After COVID-19 infection:264 [99 with long COVID	Any time: 303 [92 with long COVID]; Before COVID-19 infection:303 [92 with long COVID]; After COVID-19 infection:303 [92 with long COVID	–	–	Long duration of COVID-19 symptoms ≥4 weeks	-General: fatigue, fever; -Respiratory and heart: shortness of breath, cough, chest pain, and fast beating; -Neurological: headache, mood changes, change in smell or taste, sleep problems, difficulty in concentrating, and dizziness; -Digestive: abdominal pain, diarrhea, and nausea/anorexia; -Other: joint or muscle pain, hair loss	No. Vaccination before Omicron BA.1 infection was not associated with a clear protective effect against post-COVID condition symptoms.	21
Kuodi, 2022, Israel [26] Prospective cohort study 20 [March 2020–Nov 2021]	Pfizer/BioNTech Yes	3,388 participants (951 with COVID-19 infection [340 (36%) reported receiving a single dose of COVID-19 vaccine, and 294 (31%) reported two doses]* vs. 2,437 without COVID-19 infection	294 [167 with long COVID]	317 [217 with long COVID]	–	–	Long duration of COVID-19 symptoms ≥12 weeks	-General: fatigue, post-exertional malaise; -Respiratory and heart: shortness of breath, chest pain, and fast beating or pounding heart; -Neurological: difficult thinking or concentrating (“brain fog”), headache, change in smell or taste, dizziness or lightheadedness, and pins-and-needles feelings; -Digestive: abdominal pain, diarrhea; -Other: joint or muscle pain	Yes. Vaccination with two doses of COVID-19 vaccine reduced the most common symptoms (such as fatigue, headache, and muscle pain).	21
Marra, 2023, Brazil Case-control study 27 [Mar 2020–Jul 2022]	Pfizer/BioNTech, Janssen, AstraZeneca, Coronavac Yes	7,051 participants [3,853 (54.6%) vaccinated and 3,198 (45.4%) unvaccinated prior to COVID-19 infection; 1,933 (27.4%) reported post-COVID symptoms and 5,118 (72.6%) did not]	937 [272 with long COVID]	3,198 [1,175 with long COVID]	2,503 [388 with long COVID]	133 [2 with long COVID]	Long duration of COVID-19 symptoms ≥4 weeks	-General: fatigue, fever; -Respiratory and heart: shortness of breath, cough, chest pain, and fast beating; -Neurological: headache, mood changes, change in smell or taste, and difficulty in concentrating; -Digestive: abdominal pain, diarrhea; -Other: joint or muscle pain	Yes. COVID vaccination before infection was protective against post-COVID condition. The authors suggest that booster doses may be important in the prevention.	21
Meza-Torres, 2022, UK Retrospective cohort study 19 [Mar 2020–Sep 2021]	NR Yes [and after COVID-19 infection too]	416,505 participants had a diagnosis of COVID-19 infection, being 1.83% of those with post-COVID conditions	Any time: 305,597 [6,380 with long COVID]; Before COVID-19 infection:736 [10 with long COVID]; After COVID-19 infection:304,861 [6,370 with long COVID]	Any time: 84,966 [872 with long COVID]; Before COVID-19 infection:399,671 [7,347 with long COVID]; After COVID-19 infection:84,966 [872 with long COVID]	–	–	Long duration of COVID-19 symptoms ≥4 weeks	-General: fatigue, fever; -Respiratory and heart: shortness of breath, cough, chest pain, and fast beating; -Neurological: headache, mood changes, change in smell or taste, and difficulty in concentrating; -Digestive: abdominal pain, diarrhea; -Other: joint or muscle pain	No. No benefit of preventing post-COVID conditions.	21
Mohr, 2023, USA Prospective cohort study 9 [Dec 2020–Aug 2021]	Pfizer/BioNTech and Moderna Yes	419 participants with COVID-19, 298 (71%) reported one or more COVID-like symptoms 6 weeks after illness onset, with a lower prevalence among vaccinated participants compared with unvaccinated participants (60.6% vs 79.1%; adjusted risk ratio 0.70, 95% CI 0.58 to 0.84).	180 [109 with long COVID]	239 [189 with long COVID]	–	–	Long duration of COVID-19 symptoms ≥6 weeks	-General: fatigue, fever; -Respiratory and heart: shortness of breath, cough, chest pain, and fast beating; -Neurological: headache, mood changes, change in smell or taste, sleep problems, difficulty in concentrating, and dizziness; -Digestive: abdominal pain, diarrhea; -Other: joint or muscle pain	Yes. Receipt of two doses of a COVID-19 mRNA vaccine was associated with decreased prevalence of COVID-like symptoms at 6 weeks and earlier return to work.	21
Nehme, 2022 [1], Switzerland Cross-sectional study 3 [Apr 2021–Jul 2021]	Pfizer/BioNTech and Moderna No	1,596 participants [online survey, previously COVID-19 infection; 20,5% vaccinated with two doses]	347 [69 with long COVID]	825 [241 with long COVID]	–	–	Having any of the 6 symptoms described in the study for more than 6 months after the COVID-19 infection	-General: fatigue; -Respiratory: shortness of breath; -Neurological: difficulty concentrating or memory loss, loss of or change in smell or taste, headache;	Yes. Vaccination was associated with a decreased prevalence of post-COVID condition compared to no vaccination.	16
Perlis, 2022, USA Cross-sectional study 29 [Feb 2020–Jul 2022]	Pfizer/BioNTech, Moderna, and Janssen Yes	16,091 participants [online survey, previously COVID-19 infection; 14.7% reported post-COVID condition]	2,243 [249 with long COVID]	13,434 [2,052 with long COVID]	–	–	Long duration of COVID-19 symptoms ≥8 weeks	NR	Yes. Completion of the primary vaccine series prior to COVID-19 infection was associated with diminished risk for post-COVID condition (OR, 0.72; 95% CI, 0.60–0.86).	21
Pinato, 2022, UK Retrospective cohort study 22 [Feb 2020–Nov 2021]	Pfizer/BioNTech, Moderna, AstraZeneca, and Janssen Yes	2090 participants with COVID-19 infection (1,930 unvaccinated, 91 fully vaccinated, and 69 partially vaccinated)	60 [4 with long COVID]	1,135 [195 with long COVID]	–	–	NR	-General: fatigue, weight loss -Respiratory: dyspnea and cough) -Neurological: neuro-cognitive sequelae (cognitive, visual impairment, ano/dysosmia, age/dysgeusia, headache, confusion, and lethargy) Other: organ dysfunctions, residual fever, muscle cramps, arthralgia, and skin conditions	Yes. The additional analysis performed among COVID-19 survivors who underwent a clinical reassessment at participating centers suggests that the protection provided by vaccines extends beyond the acute phase, as supported by the reduced incidence of sequelae in fully vaccinated patients.	20
Richard, 2023, USA Retrospective cohort study 22 [Feb 2020–Dec 2021]	Pfizer/BioNTech, Moderna, and Janssen Yes [and after COVID-19 infection too]	3,363 participants with COVID-19 infection submitted surveys 1, 3, 6, 9, and 12 months after the symptoms of COVID-19 infection	419 [23 with long COVID]	1,413 [121 with long COVID]	–	–	Long duration of COVID-19 symptoms ≥4 weeks	-General: fatigue -Respiratory: dyspnea and cough) -Neurological: neuro-cognitive sequelae (cognitive, visual impairment, ano/dysosmia, age/dysgeusia, headache, confusion, and lethargy) Other: organ dysfunctions, residual fever, muscle cramps, and arthralgia	Yes. Participants who were vaccinated prior to COVID-19 infection were significantly less likely to report 28 or more days of illness. Vaccination after COVID-19 infection was also associated with a lower risk of reporting symptoms at 6 months after symptom onset.	22
Selvaskandan, 2022, UK Retrospective cohort study 1 [Mar 31^st^, 2021–May 1st, 2021]	NR NR	423 participants (online questionnaire at the UK nephrology workforce)	340/363 (86%) received 2nd dose [NR absolute numbers of long COVID in vaccinated group]	21 [NR absolute numbers of long COVID in unvaccinated group]	–	–	Long duration of COVID-19 symptoms ≥12 weeks	-General: fatigue, post-exertional malaise; -Respiratory and heart: shortness of breath, cough, and fast beating or pounding heart; -Neurological: difficult thinking or concentrating (“brain fog”), headache, mood changes, change in smell or taste, and sleep problems; -Other: joint or muscle pain	NR	13
Senjam, 2022, India Cross-sectional study 6 [Jan 2021–Apr 2021, and June/July 2021]	NR Yes	773 of 1,081 participants responded to the online questionnaire (42.9%). One-third of the participants reported having post-COVID condition symptoms.	191 [50 with long COVID]	407 [142 with long COVID]	–	–	Long duration of COVID-19 symptoms ≥4 weeks	-General: fatigue, post-exertional malaise; -Respiratory and heart: shortness of breath, cough, and fast beating or pounding heart; -Neurological: difficult thinking or concentrating (“brain fog”), headache, mood changes, change in smell or taste, sleep problems, and dizziness; -Other: joint or muscle pain	Yes. Two doses of COVID-19 vaccine reduced the development of post-COVID condition.	22
Tannous, 2023, USA Retrospective cohort study 21 [Mar 2020–Nov 2021]	Pfizer/BioNTech, Moderna, AstraZeneca, and Janssen Yes	53,239 participants with COVID-19 infection (49,458 unvaccinated and 3,781 fully vaccinated)	3,781 [332 with long COVID]	49,458 [5,597 with long COVID]	–	–	Long duration of COVID-19 symptoms ≥4 weeks	Constitutional: palpitations, malaise/fatigue, and headache Systemic: sleep disorders, shortness of breath, mood/anxiety disorders, cough, and cognitive impairment	Yes. The likelihood of long COVID reduction was consistently greater among vaccinated breakthrough COVID-19 cases.	21
Taquet, 2022, UK[27] Retrospective cohort study 8 [Jan 2021–Aug 2021]	Pfizer/BioNTech, Moderna, and Janssen Yes	10,024 participants with COVID-19 infection recorded at least 2 weeks after the first dose of COVID-19 vaccine vs. 83,957 unvaccinated (after propensity scored matching: 9,479 were matched to unvaccinated participants)	6,957 [4,459 with long COVID]	6,957 [4,512 with long COVID]	–	–	6-month incidence of all Long COVID outcomes. ICD-10 codes were used detect the post-COVID conditions	General: fatigue; -Respiratory and heart: shortness of breath, chest pain; -Neurological: difficult thinking or concentrating (“brain fog”), headache, mood changes, sleep problems, and pins-and-needles feelings; -Digestive: abdominal pain; -Other: joint or muscle pain	No. Receiving two doses of COVID-19 vaccine was not associated with a lower risk of long COVID-19.	21
Thaweethai, 2023, USA Prospective cohort study 17 [Dec 2021–Apr 2023]	Pfizer/BioNTech, Moderna, and Janssen Yes	9,764 participants in the RECOVER adult cohort [2231 participants first COVID-19 infection on or after December 1, 2021, and enrolled within 30 d of infection, 224 (10%) had post-COVID condition at 6 mo].	2,016 [195 with long COVID]	86 [15 with long COVID]	–	–	Long duration of COVID-19 symptoms ≥6 months	-General: fatigue, post-exertional malaise; -Respiratory and heart: shortness of breath, cough, fast beating or pounding heart; -Neurological: difficult thinking or concentrating (“brain fog”), headache, mood changes, change in smell or taste, sleep problems, dizziness, tinnitus, and vision problems; -Digestive: abdominal pain, diarrhea; -Other: joint or muscle pain; changes in menstrual cycle; hair loss, rash	Yes. Vaccination with two doses of COVID-19 vaccine reduced the most common symptoms.	23
Van der Maaden, 2023, Netherlands Prospective cohort study 7 [May 2021–Dec 2021]	Pfizer/BioNTech, Moderna, AstraZeneca, and Janssen Yes	10,389 participants at 3 months of follow-up [6,614 cases, 1,330 test-negative control, and 2,245 invited population controls]	4,902 [2,322 with long COVID]	542 [271 with long COVID]	–	–	Long duration of COVID-19 symptoms ≥12 weeks	-General: fatigue, post-exertional malaise; -Respiratory and heart: shortness of breath, chest pain, cough, and fast beating or pounding heart; -Neurological: difficulty thinking or concentrating (“brain fog”), change in smell or taste; -Other: joint or muscle pain	Yes. Vaccination prior to infection was protective against loss of smell and taste in cases aged <65 years.	21
Zisis, 2022, USA Retrospective cohort study 15 [Sep 2020–Dec 2021]	NR Yes	1,578,719 participants with confirmed COVID-19 were identified (TriNetX Research Network platform), and 1.6% (*n* = 25 225) completed vaccination	25,225 [2,707 with long COVID]	25,225 [5,671 with long COVID]	–	–	Long duration of COVID-19 symptoms ≥4 weeks	-General: fatigue, post-exertional malaise; -Respiratory and heart: shortness of breath; -Neurological: difficulty thinking or concentrating (“brain fog”), headache; -Digestive: Diarrhea; -Other: joint or muscle pain	Yes. COVID-19 vaccine is protective against post-COVID condition.	22

D&B score, Downs and Black score; ICD-10, 10^th^ revision of the International Classification of Diseases; WHO, World Health Organization; NR, Not reported.

*[From Kuodi 2022 study] – At the time of data collection, very few individuals had received a third dose, and those who did were recorded as two doses.

One-quarter of the studies included in our review were conducted in the United States (8 studies).^25,36,41,43,45,48,50,51^ Seven studies were performed in the United Kingdom,^26,27,29,40,44,46,49^ three in Switzerland,^17,31,42^ two in India,^28,47^ and one of each was performed in Brazil,^39^ France,^34^ Israel,^38^ Italy,^30^ Morocco,^33^ Netherlands,^16^ Norway,^32^ Saudi Arabia,^24^ Scotland,^35^ South Africa,^37^ Spain,^15^ and Turkey.^14^ All studies were performed between February 2020 and April 2023.^14–17,24–51^ The study duration varied from 1 to 29 months.

In our qualitative analysis, thirty-two studies, including 775,931 individuals, evaluated the effect of vaccination among fully vaccinated individuals and unvaccinated individuals on post-COVID conditions.^14–17,24–51^ Twenty studies evaluated VE in individuals vaccinated only before COVID-19 infection,^14–16,25–27,29–31,36,38,39,41,43,44,47–51^ three studies evaluated VE for post-COVID conditions among those who were vaccinated after COVID-19 infection,^32,33,42^ four studies evaluated VE among those who were vaccinated before and after COVID-19 infection,^17,37,40,45^ and five studies evaluated VE but did not specify the timing of the vaccine^24,28,34,35,46^. All 32 studies evaluated VE with at least two doses of a COVID-19 vaccine.^14–16,23–51^ Three studies evaluated vaccinated individuals with three doses of vaccine.^30,31,39^ While eight of 32 studies reported data during Omicron variant era,^15,17,27,30,31,39,43,50^ 24 studies took place before Omicron variant era.^14,16,24–26,28,29,32–38,40–42,44–49,51^

Each study adopted different definitions for post-COVID conditions (Table 1). Post-COVID conditions were defined as symptoms lasting more than 4 weeks in thirteen studies,^15,17,25–28,30,39,40,45,47,48,51^ more than 12 weeks in nine studies,^16,29,32,33,35–38,46^ and more than 6 months in seven studies.^14,24,31,34,42,49,50^. One study defined symptoms as more than 6 weeks,^41^ one study defined symptoms as more than 8 weeks as a post-COVID condition^43^, and one study did not report the duration of symptoms.^44^ All studies used at least one of the common symptoms (details shown in Table 1) to make a diagnosis of a post-COVID condition.^14–16,23–51^ Nearly three-quarters of the included studies (22 studies) showed that vaccination was protective against post-COVID symptoms.^14,16,25–27,29–32,35,36,38,39,41–45,47,48,50,51^ Nine studies did not report any benefit of COVID-19 vaccination in reducing post-COVID condition symptoms,^15,17,24,28,33,34,37,40,49^ and one study did not report any statistical analysis of effectiveness.^46^

**Figure 2.**
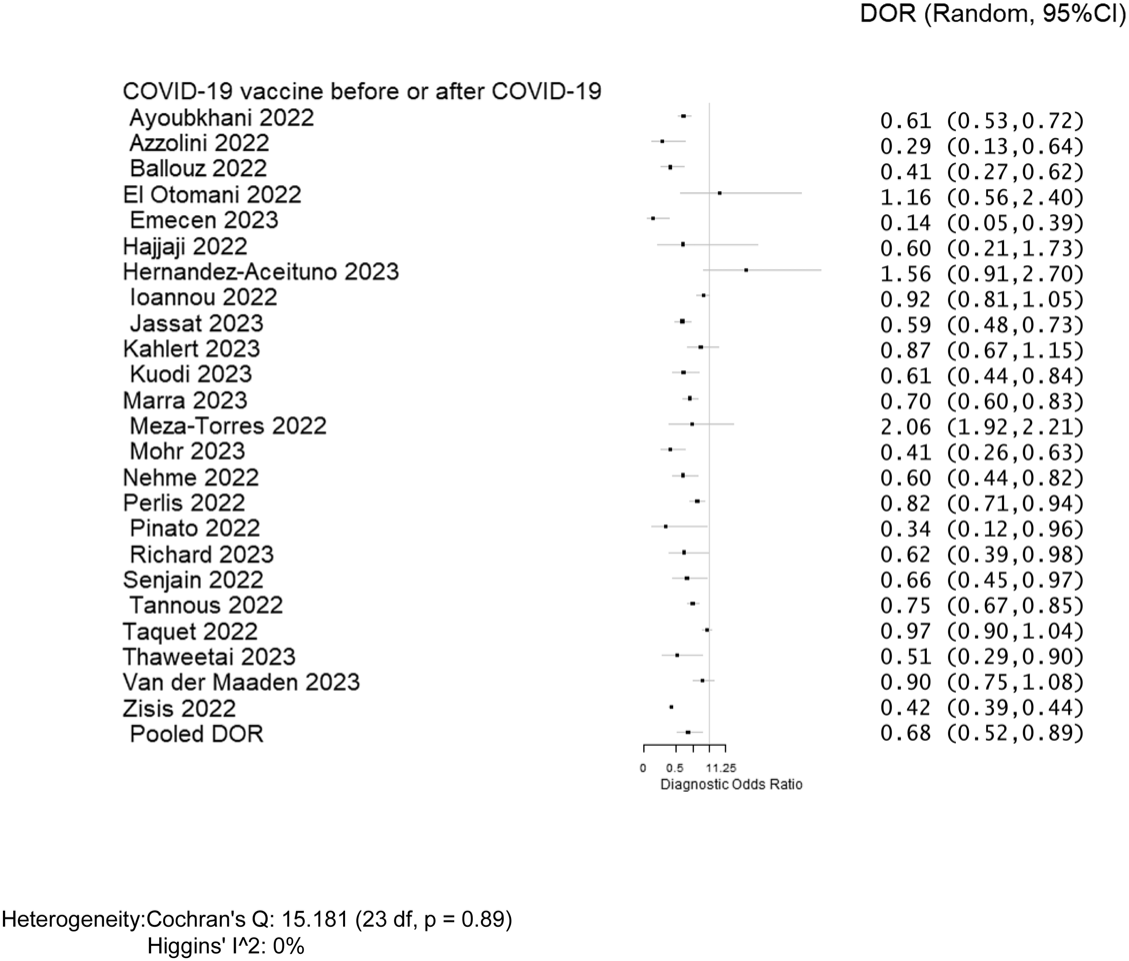
Forest plot of COVID-19 vaccine effectiveness among post-COVID conditions in individuals who received COVID-19 vaccine before or after COVID-19 infection. Diagnostic odds ratios (DOR) were determined with the DerSimonian and Laird random-effects method. Abbreviation: CI, confidence interval.

Overall, twenty-four studies, including 620,221 individuals, evaluated post-COVID conditions among those who received at least two doses of COVID-19 vaccine before or after COVID-19 infection (Table 2) and were included in the meta-analysis.^14–17,29–31,33,34,36–45,47–51^ The pooled prevalence of post-COVID conditions was 11.8% among those who were unvaccinated and 5.3% among those who received at least two doses. The pooled DOR for post-COVID conditions among individuals vaccinated with two doses was 0.680 (95% CI: 0.523–0.885) with an estimated VE of 32.0% (95% CI: 11.5%–47.7%) (Figure 2). Of the twenty-four studies, twenty-one evaluated post-COVID conditions in individuals who received the COVID-19 vaccine before infection.^14–17,29–31,36–41,43–45,47–51^ The DOR was 0.631 (95% CI: 0.518–0.769) (Supplementary Appendix 2), and the estimated VE was 36.9% (95% CI: 23.1%–48.2%) (Table 2). There were five papers that evaluated post-COVID conditions for those who received the vaccine after infection.^17,33,37,40,42^ The DOR was 1.303 (95% CI: 0.890–1.907) (Supplementary Appendix 3), and it was not possible to estimate VE because it did not prevent post-COVID condition (Table 2). There were seven studies that evaluated post-COVID conditions for those who received the COVID-19 vaccine only before infection during the Omicron variant era.^15,17,30,31,39,43,50^ The DOR was 0.684 (95% CI: 0.542–0.862) (Supplementary Appendix 4), and the estimated VE was 31.6% (95% CI: 13.8%–45.8%) (Table 2). There were three studies that evaluated post-COVID conditions for those who received the additional booster dose vaccine only before infection.^30,31,39^ The DOR was 0.313 (95% CI: 0.278–0.353) (Supplementary Appendix 5), and the estimated VE was 68.7% (95% CI: 64.7%–72.2%) (Table 2). Because there were no studies evaluating post-COVID conditions for those who received two doses of each specific type of COVID-19 vaccine (mRNA or viral vector or inactivated viral vaccine), we did not perform a stratified analysis. The results of meta-analyses were homogeneous for studies evaluating post-COVID conditions in individuals who received the COVID-19 vaccine before or after COVID-19 infection (heterogeneity *p* = 0.89, *I* ^2^ = 0%), and homogenous for studies evaluating post-COVID conditions in individuals receiving vaccine before infection (heterogeneity *p* = 0.62, *I* ^2^ = 0%), and also homogenous for studies evaluating post-COVID conditions in individuals receiving vaccine after infection (heterogeneity *p* = 0.29, *I* ^2^ = 19.9%), respectively. There was no evidence for publication bias among the 24 studies included in the meta-analysis^14–17,29–31,33,34,36–45,47–51^ (*p* = 0.71).

**Table 2.** Subset analyses evaluating COVID-19 vaccine effectiveness among post-COVID conditions in individuals who received COVID-19 vaccine before or after COVID-19 infection

Vaccinated individuals	COVID-19 vaccine before/after COVID-19 infection**	Studies included (*n*)	Participants [vaccinated + unvaccinated] (*n*)	Pooled Diagnostic Odds Ratio [DOR] (95% CI)	I^2^ test for heterogeneity	Vaccine effectiveness* (95% CI)
Fully vaccinated	Before/After	24	620,221	0.680	0%	32.0%
				(0.523, 0.885)		(11.5%, 47.7%)
Fully vaccinated	Before	21	618,841	0.631	0%	36.9%
				(0.518, 0.769)		(23.1%, 48.2%)
Fully vaccinated	After***	5	396,101	1.303	19.9%	–
				(0.890, 1.907)		
Fully vaccinated	Before (Omicron era)	7	25,414	0.684	50.1%	31.6%
				(0.542, 0.862)		(13.8%, 45.8%)
Booster dose (1^st^)	Before	3	5,948	0.313	0%	68.7%
				(0.278, 0.353)		(64.7%, 72.2%)

CI, Confidence Interval.

*Vaccine Effectiveness was estimated as 100% × (1-DOR).

**There is overlapping (VE for post-COVID condition who got COVID-19 vaccine before and after COVID-19 infection) in three of the studies [Jassat 2023, Kahlert 2023 and Meza-Torres 2022].^17,37,40^

***Imbalance of studies: one of the five studies with 390,563 participants [Mezza-Torres 2022]^40^, representing 98.6% of the total participants from the COVID-19 vaccine studies after COVID-19 infection.

Regarding the quality assessment scores of the 32 included studies, more than three-quarters of the studies (28 studies) were considered good quality (19–23 of 28 possible points) as per the Downs and Black quality tool.^14–17,25–32,34–41,43–45,47–51^ Three studies were considered fair (14–18 points),^24,33,42^ and one study was considered to be of poor quality (≤13 points).^46^

## Discussion

This systematic literature review and meta-analysis suggest that the pooled prevalence of post-COVID conditions was 11.8% among those unvaccinated and 5.3% among those individuals fully vaccinated. The VE of fully vaccinated individuals against post-COVID conditions was not high at approximately 30%; however, the prevalence of post-COVID conditions was lower with a statistically significant difference in fully vaccinated individuals. The stratified analysis showed a significant reduction in post-COVID conditions during the Omicron variant era, and the vaccine should be offered to unvaccinated individuals who have not had COVID-19 yet. Given VE against post-COVID conditions increased with an additional dose of the COVID-19 vaccine, individuals who have not received a booster should be encouraged to get one. There was no protection against post-COVID conditions observed if COVID-19 vaccines were given after COVID-19 infection.

With the ongoing COVID-19 pandemic, a considerable proportion of individuals who have recovered from COVID-19 infection have long-term symptoms involving multiple organs and systems.^52,53^ The percentage of individuals who have had COVID and reported post-COVID condition symptoms declined from 19% in June 2022 to 11% in January 2023^8^. A systematic review including 57 studies reported that more than half of COVID-19 survivors experienced persistent post-COVID condition symptoms 6 months after recovery.^53^ The present systematic review showed a relatively low prevalence of post-COVID conditions; this is likely because most individuals included in our studies were non-hospitalized individuals and we are presently in the Omicron variant era.^17,30,31,37,39,50,54^ Previous studies suggested that the Delta and Omicron variants caused less systemic inflammatory processes, severe illness, or death, resulting in less severe long COVID symptoms than the wild-type variant (Wuhan).^27,55^

Currently, there are no standardized criteria for diagnosing and categorizing post-COVID conditions. Another recent systematic literature review found substantial heterogeneity in defining post-COVID conditions in the published studies, with almost two-thirds (65%) not complying with the definitions from the CDC, the UK National Institute for Health and Care Excellence (NICE), or WHO (World Health Organization).^56^ This variability in definitions across studies can affect the comparability and generalizability of findings. The studies included in our systematic review used a variety of symptoms and durations to make a diagnosis of post-COVID conditions. The most common symptoms described were fatigue or muscle weakness, persistent muscle pain, anxiety, memory problems, sleep problems, and shortness of breath.^52,53,57^ Another study reported that, regardless of the initial disease severity, COVID-19 survivors had longitudinal improvements in physical and mental health, with most returning to their original work within 2 years.^52^ However, survivors had a remarkably lower health status than the general population at 2 years.^52^ The CDC reports that individuals with post-COVID conditions may experience many symptoms that can last more than 4 weeks or even months after infection and the symptoms may initially resolve but subsequently recur.^7^ This differs from the WHO definition where post-COVID conditions are defined to occur in individuals who have a history of probable or confirmed SARS-CoV-2 infection; usually within 3 months from the onset of COVID-19, with symptoms and effects that last for at least 2 months.^58^ A clearer and more standardized definition of post-COVID conditions is needed for researchers to investigate the true prevalence among those who are vaccinated and unvaccinated, and to evaluate the VE against post-COVID conditions.

While our previous meta-analysis on the same topic, but with a single vaccine dose instead of two doses, suggested that COVID-19 vaccines might effectively prevent post-COVID conditions even if administered after a COVID-19 infection,^9^ the present meta-analysis did not demonstrate any protective effect when vaccines were given after COVID-19 infection. There are a few potential reasons for this discrepancy. Our previous meta-analysis^9^ included three papers, including two preprint papers. However, none of these three papers met the inclusion criteria for the present study (two preprint papers and one paper focusing on a single vaccine). Instead, the present study included five new papers, one of which reported protection while the other four did not. It is important to interpret this stratified analysis cautiously, as one of the four studies without protection had a considerably larger sample size of over 390,000 individuals, accounting for more than 98% of the total sample size in the stratified analysis.^40^ Further studies will be necessary to investigate the effect of COVID-19 vaccines against post-COVID conditions when administered after a COVID-19 infection.

Our study had several limitations. First, the majority of the included studies in the meta-analysis investigating the VE in preventing post-COVID conditions employ different observational study designs, including cohort studies, and case-control studies.^14,17,29–31,33,34,36–42,44,45,47–51,54^ Second, features such as age, underlying health conditions, immunosuppression status, and prior COVID infection history can influence both VE and the likelihood of developing post-COVID conditions. Controlling these confounding features can be challenging in observational studies, and residual confounding factors may impact the accuracy of the estimates. Third, studies on VE for post-COVID conditions may focus on specific populations (e.g., hospitalized individuals), or geographical areas, which may limit the generalizability of the findings to other settings or populations. Fourth, VE against post-COVID conditions may change over time due to the emergence of new variants, waning immunity, or the need for additional booster doses. Understanding the dynamics of VE and the potential impact of these time-dependent effects is an ongoing area of research. Fifth, we could not find any studies that evaluated the impact of a second booster dose or bivalent vaccines on VE against post-COVID conditions. Additionally, since the definition of post-COVID conditions varies significantly over the included studies, overdiagnosis and misdiagnosis could be present. Lastly, the abstract screening was performed by one reviewer (ARM), while the review of articles was conducted independently by two individuals.

In conclusion, receiving two doses of the COVID-19 vaccine prior to COVID-19 infection significantly reduces the risk of developing post-COVID conditions compared to those who are unvaccinated during the study period, including the Omicron variant era. Vaccine effectiveness against post-COVID conditions was higher when a third dose was administered. However, no protection against post-COVID conditions was observed with vaccinations given after a person had already contracted COVID-19. More observational studies are needed to evaluate bivalent COVID-19 vaccines, vaccination after COVID-19 infection, VE of a second booster dose, VE of mixing COVID-19 vaccines, and genomic surveillance for better understanding of VE against post-COVID conditions. A more standardized definition of post-COVID conditions is still needed both for research and clinical purposes.

## Supplementary material

For supplementary material accompanying this paper visit http://doi.org/10.1017/ash.2023.447. click here to view supplementary material
