# Supplementary material for: The effectiveness of COVID-19 vaccine in the prevention of post-COVID conditions: a systematic literature review and meta-analysis of the latest research
Source: Antimicrob Steward Healthc Epidemiol. 2023 Oct 13;3(1):e168. doi: 10.1017/ash.2023.447 (PMC10644173; doi:10.1017/ash.2023.447)
Supplement: Supplementary file 1 [file S2732494X23004473sup001.zip › S2732494X23004473sup005.docx]

| #1 Long Covid | **#2 Vaccination** | **#3 Efficacy** |
| --- | --- | --- |
| MeSH  Post-Acute COVID-19 Syndrome  COVID-19/Complications | MeSH  Vaccination  COVID-19 Vaccines | MeSH  Vaccine efficacy  Disease progression  Hospitalization |
| CINAHL headings  Post-Acute COVID-19 Syndrome  COVID-19/Complications | CINAHL headings  Immunization  COVID-19 Vaccines | CINAHL headings  Vaccine efficacy  Disease progression  Hospitalization |
| Emtree  Long Covid  Coronavirus 19 with complications subheading | Emtree  Immunization  SARS-CoV-2 vaccine | Emtree  Hospitalization  Disease exacerbation  Risk factor |
| Keywords  Post acute sequelae  PASC  Post acute COVID  Postacute COVID  Long COVID  Chronic COVID  Post COVID  Covid complications  chronic  long  expanded  extended  recurr*  sustain*  persist*  prolong*  continu*  debilitating  AND  COVID  COVID 19  SARS CoV 2  Coronavirus  Corona virus  2019 nCOV | Keywords  Vaccinated  Vaccination  Vaccine  Vaccinated  Unvaccinated  immunization(s)  variolation(s)  Immunologic stimulation  Immunostimulation | Keywords  Protect* (ive, ion)  Prevent* (s, ion, ing)  Associat (ed, ion, ions)  Correlation*  Efficacy  Effect*  Improve* |

**PubMed 6/2/23**

#1

"Post-Acute COVID-19 Syndrome"[Mesh] OR "COVID-19/complications"[Mesh] OR PASC [Title/Abstract] OR Post acute COVID [Title/Abstract] OR Postacute COVID[Title/Abstract] OR "long covid"[Title/Abstract:~4] OR "chronic covid" [Title/Abstract:~4] OR "persistent covid" [Title/Abstract:~4] OR "covid complications"[Title/Abstract:~4] OR "post covid"[Title/Abstract:~4]

OR

(COVID [Title/Abstract] OR Covid 19 [Title/Abstract] OR Corona virus [Title/Abstract] OR Coronavirus [Title/Abstract] OR 2019-nCoV [Title/Abstract] OR SARS-CoV-2 [Title/Abstract] OR

2019-nCoV[Title/Abstract]) AND (long duration [Title/Abstract] OR persistent symptoms [Title/Abstract] OR post acute sequelae [Title/Abstract] OR postacute sequelae[Title/Abstract])

#2

"Immunization"[Mesh] OR "COVID-19 Vaccines"[Mesh] OR vaccin*[Title/Abstract] OR unvaccinated[Title/Abstract] OR immunization*[Title/Abstract] OR variolation*[Title/Abstract] OR "Immunologic stimulation"[Title/Abstract] OR Immunostimulation[Title/Abstract]

#3

"Vaccine Efficacy"[Mesh] OR "Disease Progression"[Mesh] OR "Hospitalization"[Mesh] OR protect*[Title/Abstract] OR prevent*[Title/Abstract] OR associat*[Title/Abstract] OR correlat*[Title/Abstract] OR efficacy[Title/Abstract] OR effect*[Title/Abstract] OR improve*[Title/Abstract]

#1 AND #2 AND #3, with pub dates 2019-2023 and editorials and commentaries excluded= 2746

**CINAHL 6/2/23**

#1

(MH "Post-Acute COVID-19 Syndrome") OR (MH "COVID-19+/CO") OR TI ( "Post acute sequelae" OR "Post acute COVID" OR PASC OR "Postacute COVID") OR AB ( "Post acute sequelae" OR "Post acute COVID" OR PASC OR "Postacute COVID") OR (chronic OR long OR expanded OR extended OR recurr* OR sustain* OR persist* OR prolong* OR continu* OR debilitating) N3 (covid OR coronavirus OR "corona virus" OR Sars Cov 2 OR 2019 nCOV)

#2

TI ( Vaccinat* OR Unvaccinated OR vaccine* OR immunization* OR variolation* OR "Immunologic stimulation" OR Immunostimulation) OR AB (Vaccinat* OR Unvaccinated OR vaccine* OR immunization* OR variolation* OR "Immunologic stimulation" OR Immunostimulation)

OR

MH "Immunization+" OR MH "COVID-19 Vaccines"

#3

MH "Disease Progression+" OR MH "Vaccine Efficacy" OR MH "Hospitalization+" OR protect* OR prevent* OR associat* OR correlat* OR efficacy OR effect* OR improve*

#1 AND #2 AND #3=513, with pub dates 2019-2023 and editorials excluded

**Embase 6/2/23**

#1

'coronavirus disease 2019'/exp/dm_co OR 'long covid'/exp OR 'post acute sequelae':ab,ti OR 'post acute covid':ab,ti OR pasc:ab,ti OR 'postacute covid':ab,ti OR (long OR chronic OR persistent OR recurrent OR prolonged) NEAR/2 (covid OR coronavirus OR 'corona virus' OR 'sars cov 2' OR '2019 ncov')

#2

'immunization'/exp OR 'sars-cov-2 vaccine'/exp OR vaccinat*:ti,ab OR unvaccinated:ti,ab OR vaccine*:ti,ab OR immunization*:ti,ab OR variolation*:ti,ab OR 'immunologic stimulation':ti,ab OR immunostimulation:ti,ab

#3

'hospitalization'/exp OR 'disease exacerbation'/exp OR 'risk factor'/exp OR protect*:ab,ti OR prevent*:ab,ti OR associat*:ab,ti OR correlat*:ab,ti OR efficacy:ab,ti OR effect*:ab,ti OR improve*:ab,ti

#1 AND #2 AND #3 with pub dates 2019-2023 and editorial excluded= 1045

**Web of Science 6/2/23**

TS=(vaccine* OR vaccination*OR pfizer OR moderna OR immunization* OR  variolation* OR  "immunologic stimulation"OR immunostimulation  OR  gamaleya  OR  "Sputnik V" OR sinovac OR "Corona Vac" OR astrazeneca OR janssen OR "AZD1222"  OR "mRNA-1273" OR janssen OR "Johnson & Johnson" OR "JNJ-78436735")

#2

TS=(((long NEAR/3 covid) OR (chronic NEAR/3 covid) OR (recurrent NEAR/3 covid) OR (persistent NEAR/3 covid) OR (postacute NEAR/3 covid) OR (post acute NEAR/3 covid) OR PASC OR post acute sequelae))

#3

TS= (success* OR prevent* OR protect* OR protect* OR prevent* OR associat* OR correlat* OR efficacy OR effect* OR improve* OR outcome* OR hospital* OR progression)

#1 AND #2 AND #3, with pub dates 2019-2023 and exclude editorial=489

**Cochrane CENTRAL 6/2/23**

**
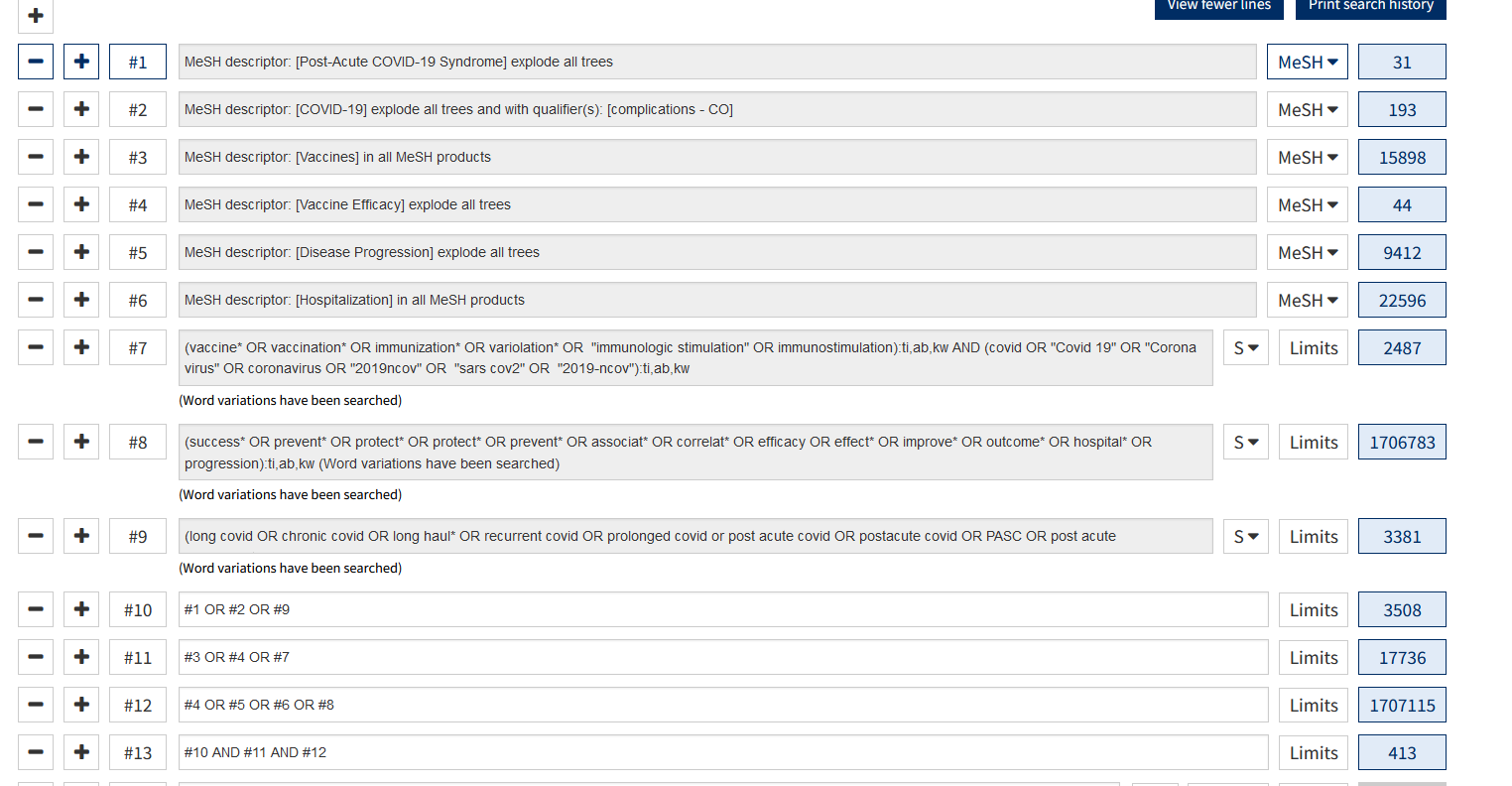
**

**=407 limited to trials**

**Scopus 6/2/23**

Citing paper and ref list search for previously included 2021 and 2022 papers=317
